# Natural history of burnout, stress, and fatigue in a pediatric resident cohort over three years

**DOI:** 10.1080/10872981.2020.1815386

**Published:** 2020-09-08

**Authors:** Lindsay R. Koressel, Elizabeth Groothuis, Robert R. Tanz, Hannah L. Palac, Sandra M. Sanguino

**Affiliations:** a Pediatrics, Division of Hospital Based Medicine, Ann & Robert H. Lurie Children’s Hospital of Chicago and Northwestern University Feinberg School of Medicine, Chicago, IL, USA; b Pediatrics, Division of Academic General Pediatrics and Primary Care, Ann & Robert H. Lurie Children’s Hospital of Chicago and Northwestern University Feinberg School of Medicine, Chicago, IL, USA; c Independent Statistical Consultant, Milwaukee, WI, USA; d Medical Education, Northwestern University Feinberg School of Medicine, Chicago, IL, USA; e Pediatrics, Division of Academic General Pediatrics and Primary Care, Ann & Robert H. Lurie Children’s Hospital of Chicago Pediatrics, Division of Academic General Pediatrics and Primary Care, Chicago, IL, USA

**Keywords:** Residency training, burnout, stress, fatigue, resilience, social connectedness, medical education

## Abstract

**Background:**

Burnout is known to be high amongst physician trainees. Factors such as stress, fatigue, social environment, and resilience could affect burnout. Cross-sectional data describe burnout in pediatric residents, but the trajectory of burnout in a cohort of residents followed longitudinally through the full course of residency training has not been reported.

We prospectively examined the prevalence and trajectory of burnout, stress, fatigue, social connectedness, and resilience in a pediatric resident cohort from orientation through three years of residency.

The cohort (N = 33) was surveyed six times between 2015–2018 using the Abbreviated Maslach Burnout Inventory (AMBI), Perceived Stress Scale (PSS), Epworth Sleepiness Scale (ESS), Social Connectedness Scale-Revised (SCS-R), and Connor-Davidson Resilience Scale (CD-RISC10). Data were analyzed using repeated measures mixed effects models. Significant change from baseline was considered to be adjusted p < 0.05.

Response rate was >50% at each timepoint; 69% of trainees completed surveys ≥4 times. Scores were significantly worse than baseline in all surveys, at every timepoint, with the exception of AMBI-PA (personal accomplishment) at the PGY1/PGY2 transition and SCS-R and CD-RISC10 at the end of training. The most significant changes from baseline occurred mid-PGY1 to mid-PGY2. At least 65% of residents demonstrated worse scores than baseline on 36/40 (90%) follow-up surveys. Furthermore, ≥65% met criteria for emotional exhaustion and moderate stress at every timepoint. SCS-R was the only survey measure to improve at residency completion compared to baseline.

**Conclusion:**

Within 6 months of starting residency this pediatric resident cohort became burned out, stressed, fatigued, less socially connected, and less resilient. Burnout is only one factor that indicates impaired resident well-being. To fully address this, a comprehensive examination of how residents are trained is needed to identify effective interventions.

**Abbreviations:**

MBI – Maslach Burnout Inventory; AMBI – Abbreviated Maslach Burnout Inventory; AMBI-EE – Emotional Exhaustion; AMBI-D – Depersonalization; AMBI-PA – Personal Accomplishment; AMBI-SAT – Satisfaction with Medicine; LCH – Ann & Robert H. Lurie Children’s Hospital of Chicago/Lurie Children’s Hospital; P/CN – Pediatrics/Child Neurology; PSS – Perceived Stress Scale; ESS – Epworth Sleepiness Scale; CD-RISC10 – Resilience; SCS-R – Social Connectedness Scale Revised; PGY – Post-Graduate Year

## Introduction

Burnout is an undesirable state of mental and physical exhaustion related to work [1]. Burnout is more common amongst medical professionals than other workers and college graduates [2,3]. Burnout has been shown to increase during medical school and can persist in residents through training [4,5]. Multiple studies have demonstrated that patient care [1,6] and self-reported medical errors [7] can be negatively impacted by burnout. Given its prevalence in the medical profession, developing interventions to mitigate burnout and identifying factors associated with resilience (to counter burnout) have become priorities in post-graduate medical training [8].

The validated tools most frequently used to measure burnout are the Maslach Burnout Inventory (MBI) and the Abbreviated MBI (AMBI) [9–11]. Burnout is characterized by emotional exhaustion (EE), feeling emotionally overextended and exhausted at work; depersonalization (D), an unfeeling or impersonal response toward recipients of one’s care; and decreased sense of personal accomplishment (PA), feeling less competent and successful at work [10,11]. Factors that could contribute to burnout include large clinical workload, stressful work environment, sleep deprivation and fatigue, and unpleasant social or work environment. Personal resilience, the ability to withstand or recover from adverse experiences, could protect against burnout. Studies have assessed burnout, workload, and sleep in pediatric residents [5,12,13], but there have not been longitudinal cohort studies of pediatric residents followed through 3 years of residency.

When burnout occurs, it must start at some time or subsequent to events or circumstances. However, there are no data that describe the trajectory of burnout in pediatric residency. It is not known if burnout, having developed, persists unabated throughout training or if there are changes over time. Does burnout generally increase or decrease during training, or does it wax and wane? Are there factors that coexist with burnout that might influence the trajectory of burnout or the development of successful interventions? Studies are needed to address the natural history of burnout in trainees so effective mitigation or prevention efforts can be planned, implemented, and maintained.

The aim of this study was to examine the prevalence and trajectory of burnout, perceived stress, fatigue, social connectedness, and resilience in a cohort of pediatric residents over the 3-year course of residency training.

## Methods

### Setting

Ann & Robert H. Lurie Children’s Hospital (LCH) is a large tertiary, academic children’s hospital affiliated with the McGaw Medical Center of Northwestern University Feinberg School of Medicine. At the time of this study, the pediatric residency program had 33 residents/year, including 1–2 residents in the combined pediatrics/child neurology (P/CN) residency.

### Survey measures

We asked residents about their age, gender, living arrangements, marital status, and recent life events as general characteristics that help describe the cohort (Table 1). We used 5 validated tools to assess trainees’ well-being, each described below.

**Table 1.** Study site, residency program, and study population baseline demographics.

Resident Baseline Demographics^a^	N	%
**Residents**	**33**	**100**
**Gender**		
Prefer not to say	1	3
Female	27	82
Male	5	15
**Race**		
Asian/Pacific Islander	4	12
Black/African-American	2	6
White/Caucasian	22	67
Multiple races	5	15
**New to Chicago**		
No	3	9
Yes	29	91
No response	1	
**Living Arrangement**		
Alone	13	39
Roommate	5	15
Spouse/Partner	15	45
**Marital Status**		
Single	21	64
Engaged	2	6
Married	9	27
Divorced	1	3
**Hospital Characteristics**		
Inpatient Admissions^b^	10,891	
ED Visits^b^	47,079	
Deliveries^c^	11,648	
**Residency Schedule**	**Inpatient**	**Outpatient/ED^d^/Elective**
PGY1	8–9 months	3–4 months
PGY2	6–7 months	5–6 months
PGY3	4–5 months	7–8 months

^a^Demographic Data from PGY1 residents at T1 (Orientation)

^b^2015–2016 data (Cohort’s PGY1 year)

^c^Calendar year 2016 deliveries at Prentice Women’s Hospital, where pediatric residents rotate through Newborn Nursery and NICU

^d^Emergency Department

The Abbreviated Maslach Burnout Inventory (AMBI) is a validated, self-administered, 12-item, one-page inventory derived from the 22-item MBI [9–11]. It assesses Emotional Exhaustion (AMBI-EE), Depersonalization (AMBI-D), and Personal Accomplishment (AMBI-PA). The maximum score for each scale is 18. Higher scores on the AMBI-EE indicate more emotional exhaustion (feelings of being emotionally overextended and exhausted at one’s work). Higher scores on the AMBI-D indicate more depersonalization (an impersonal response toward recipients of one’s care). Higher AMBI-PA scores indicate greater sense of personal accomplishment (feelings of competence and successful achievement in one’s work). We supplemented the AMBI with 3 questions about satisfaction with medicine (AMBI-SAT) that have been used previously[11]. AMBI-SAT has a maximum score of 18; higher scores indicate more satisfaction with medicine.

The Perceived Stress Scale (PSS) was chosen as a validated instrument to assess the degree to which residents experienced stress [14,15]. A PSS score of 0–13 is considered low stress, 14–26 is considered moderate stress, and 27–40 is considered high stress.

The Epworth Sleepiness Scale (ESS) was chosen as a validated 8-question survey that provides an estimate of a person’s average level of sleepiness in daily life [16,17]. It can be used as an indirect measure of sleep and sleep quality; we used it as a proxy for fatigue. ESS scores of 0–10 are considered normal, 11–12 indicates mild excessive daytime sleepiness, 13–15 indicates moderate sleepiness, and 16–24 indicates severe sleepiness.

As resilience, or a person’s ability to recover from adverse experiences, might mitigate burnout, the Connor-Davidson Resilience Scale was chosen to measure a trainee’s level of resilience [18–20]. It has been validated in a variety of populations including nurses, medical students, and physicians. We used the validated 10-item scale (CD-RISC10). CD-RISC10 has a maximum score of 40; higher scores indicate greater resilience (ability to recover from adverse experiences).

An individual’s social support system can affect the perception of their experiences and possibly impact feelings of burnout; therefore, we used the Social Connectedness Scale-Revised (SCS-R), a validated 20-item scale that measures the sense of belonging or the feeling of interpersonal closeness to others within an individual’s social world [21,22]. It is often used in studies of college students and young adults. SCS-R has a maximum score of 120 with a studied population mean of 88; higher scores indicate more social connectedness (sense of belonging or the feeling of interpersonal closeness to others within an individual’s social world).

### Survey administration

The Post-Graduate Year (PGY) cohort entering our program in June 2015 was followed through their 3-year residency. Survey names and topics were masked to prevent the participants from adjusting their responses to the subject or purpose of each scale/survey used. Surveys were administered in random order twice yearly between June 2015 and May 2018 (Table 2). Demographic data was collected every 6 months with survey administration. Distribution of the planned mid-PGY3 surveys (Time 6; T6) did not occur due to scheduling conflicts between investigators and the residency program.

**Table 2.** Survey timing and response rates.

**Timepoint**	**Description of Timepoint**	**Dates of Survey Administration**	**# Residents at timepoint**^a^	**# Residents who completed survey at timepoint**	**Response Rate**
T1	Orientation prior to PGY1	June 2015	33	33	100%
T2	Mid-PGY1	January 2016	33	26	79%
T3	PGY1/2 Transition	June 2016	33	17	52%
T4	Mid-PGY2	January 2017	33	20	61%
T5	PGY2/3 Transition	June 2017	33	23	70%
T6	Mid-PGY3	**Surveys not administered**			
T7	Completion/End of residency	June 2018	31	19	61%
**# Total surveys completed by an individual resident**		**Number of Residents**		**% of Residents**	
1^b^		3		9%	
2		4		12%	
3		3		9%	
4		6		18%	
5		8		24%	
6		9		27%	

^a^There were 33 PGY1 residents at orientation (T1). There were 31 PGY3 residents at T7. Two residents began the neurology portion of their Pediatrics/Child Neurology training after T5

^b^Residents who only completed 1 survey (baseline T1) and no other surveys were not included in the statistical analysis

The LCH Institutional Review Board determined that this study was exempt from review. The pediatric residency director approved the study. It was presented at PGY1 orientation in June 2015 as a study of how residency training affected trainees, but the terms ‘burnout,’ ‘stress,’ ‘fatigue,’ ‘resilience’, and ‘social connectedness’ were not mentioned. Residents were given the opportunity to opt out at that time and at any time throughout the study; completion of surveys was considered consent to participate. One resident opted out at the end of the PGY1 year. Surveys were distributed in person by the investigators at orientation, and thereafter online via a survey link or in person. To ensure all surveys were de-identified, the program assigned each resident a unique identification number that was unknown to the investigators. The 2 P/CN program residents were not surveyed after beginning the neurology portion of training following their PGY2 year.

### Statistical analysis

We considered the results of each survey score to be on a continuum from best to worst score. We defined each survey’s best score as the most desirable outcome and its worst score as its least desirable outcome. At each study timepoint, the mean, standard deviation, median, and quartile scores were calculated for each survey measure. Change from baseline to each timepoint was compared using repeated measures mixed effects models using an unstructured covariance matrix with the baseline value as a covariate and timepoint as the repeated measure. A random subject effect was included to account for the inherent autocorrelation between repeated measures. Comparisons were also made between the worst mean score for each survey measure and the final (T7) mean scores to evaluate the possibility of ‘recovery’ in each domain. Differences of the least-squares means for each timepoint comparison were also calculated using repeated measures mixed effects models using an unstructured covariance matrix with the timepoint as the repeated measure and a random subject effect. All subjects are included in the baseline data (Table 3, T1) but only the 30 subjects who completed more than 1 survey are included in comparison statistical models (Table 3, T2-T7). Comparisons were considered statistically significant at adjusted p < 0.05. SAS Enterprise Guide software, version 7.1 (SAS Institute, Inc. Cary, NC, USA) was used for statistical analysis.

**Table 3.** Change from baseline mean at each study timepoint.

Survey Metric^a^	Timepoint	N	Timepoint Mean	Baseline Mean^b^	Change from baseline			Model-based Statistics		
					Mean Change	Std Dev	95% CI	LS Means	Std Err	p-value
**AMBI-EE^c^**	**T1**	33	6.64	6.64						
	**T2**	26	11.46	7.04	4.42	3.79	(2.89, 5.95)	4.57	0.61	<.0001
	**T3**	17	12.94	6.65	6.29	3.39	(4.55, 8.04)	4.94	0.74	<.0001
	**T4**	20	11.05	6.75	4.30	4.18	(2.34, 6.26)	4.29	0.68	<.0001
	**T5**	23	11.39	6.70	4.70	5.09	(2.49, 6.90)	4.69	0.68	<.0001
	**T7**	19	11.63	6.53	5.11	4.48	(2.94, 7.27)	5.12	0.73	<.0001
**AMBI-D^d^**	**T1**	33	2.55	2.55						
	**T2**	26	6.15	2.19	3.96	3.47	(2.56, 5.36)	3.95	0.65	<.0001
	**T3**	17	7.53	2.41	5.12	3.02	(3.57, 6.67)	4.83	0.73	<.0001
	**T4**	20	6.00	2.25	3.75	3.01	(2.34, 5.16)	3.70	0.56	<.0001
	**T5**	23	6.39	2.17	4.22	4.08	(2.45, 5.98)	3.82	0.78	<.0001
	**T7**	19	7.26	2.42	4.84	4.38	(2.73, 6.95)	4.92	0.89	<.0001
**PSS^e^**	**T1**	33	12.52	12.52						
	**T2**	26	19.85	11.77	8.08	5.70	(5.78, 10.38)	8.17	1.06	<.0001
	**T3**	17	19.82	12.00	7.82	5.23	(5.13, 10.52)	7.65	1.09	<.0001
	**T4**	20	18.35	11.50	6.85	6.13	(3.98, 9.72)	7.07	1.10	<.0001
	**T5**	23	17.00	10.91	6.09	5.70	(3.62, 8.55)	5.45	1.07	<.0001
	**T7**	19	19.32	11.68	7.63	6.09	(4.69, 10.57)	7.91	1.32	<.0001
**ESS^f^**	**T1**	33	7.47	7.47						
	**T2**	26	10.77	7.71	3.06	3.03	(1.83, 4.28)	3.03	0.60	<.0001
	**T3**	17	11.18	7.94	3.24	3.40	(1.49, 4.98)	3.14	0.71	0.0001
	**T4**	20	11.55	7.63	3.93	2.63	(2.70, 5.15)	4.46	0.61	<.0001
	**T5**	23	10.74	7.28	3.46	3.72	(1.85, 5.07)	2.95	0.75	0.0005
	**T7**	18	10.78	6.81	3.97	3.19	(2.38, 5.56)	3.77	0.62	<.0001
**AMBI-PA^g^**	**T1**	33	14.77	14.77						
	**T2**	26	14.31	15.13	−0.83	2.23	(−1.73, 0.07)	−0.82	0.40	0.0468
	**T3**	17	14.62	15.18	−0.56	2.14	(−1.66, 0.54)	−0.25	0.33	0.4535
	**T4**	20	13.80	15.28	−1.48	2.19	(−2.50, −0.45)	−1.28	0.41	0.0044
	**T5**	23	13.74	15.28	−1.54	2.27	(−2.53, −0.56)	−1.53	0.37	0.0002
	**T7**	19	13.63	15.08	−1.45	2.74	(−2.77, −0.13)	−1.43	0.47	0.0049
**AMBI-SAT^h^**	**T1**	33	14.73	14.73						
	**T2**	26	6.77	14.88	−8.12	3.66	(−9.59, −6.64)	−8.25	0.49	<.0001
	**T3**	17	7.59	15.06	−7.47	4.56	(−9.81, −5.13)	−7.55	0.62	<.0001
	**T4**	20	13.30	14.85	−1.55	2.48	(−2.71, −0.39)	−2.07	0.67	0.0042
	**T5**	23	12.30	15.13	−2.83	4.03	(−4.57, −1.08)	−2.75	0.70	0.0005
	**T7**	19	11.42	15.11	−3.68	4.19	(−5.70, −1.66)	−3.10	0.78	0.0004
**CD-RISC10^i^**	**T1**	33	30.61	30.61						
	**T2**	26	28.77	30.42	−1.65	3.32	(−3.00, −0.31)	−1.71	0.61	0.0089
	**T3**	17	29.24	31.59	−2.35	3.00	(−3.89, −0.81)	−1.98	0.66	0.0060
	**T4**	20	28.25	30.35	−2.10	3.70	(−3.83, −0.37)	−2.03	0.69	0.0069
	**T5**	23	28.00	30.96	−2.96	4.20	(−4.77, −1.14)	−2.94	0.79	0.0009
	**T7**	18	29.50	30.72	−1.22	5.14	(−3.78, 1.33)	−1.22	1.19	0.3172
**SCS-R^j^**	**T1**	33	99.85	99.85						
	**T2**	26	93.27	100.69	−7.42	9.51	(−11.26, −3.58)	−7.30	1.80	0.0004
	**T3**	17	92.41	102.12	−9.71	11.43	(−15.58, −3.83)	−6.58	2.34	0.0089
	**T4**	20	95.20	101.55	−6.35	9.89	(−10.98, −1.72)	−6.00	1.92	0.0041
	**T5**	23	92.22	101.17	−8.96	12.68	(−14.44, −3.48)	−9.17	2.63	0.0017
	**T7**	18	100.83	103.94	−3.11	7.20	(−6.69, 0.47)	−5.23	1.44	0.0012

^a^See text for abbreviations and descriptions; References [9–11,14–22]

^b^Baseline means at each timepoint were calculated for subjects who provided responses at the follow-up timepoint

^c^AMBI-EE: Maximum score for subscale is 18; higher scores indicate feeling more emotional exhaustion

^d^AMBI-D: Maximum score for subscale is 18, higher scores indicate feeling more depersonalization

^e^PSS: 0–13 low stress, 14–26 moderate stress, 27–40 high stress

^f^ESS: 0–10 normal, 11–12 mild sleepiness, 13–15 moderate sleepiness, 16–24 severe sleepiness

^g^AMBI-PA: Maximum score for subscale is 18, lower scores indicate feeling less personal accomplishment

^h^AMBI-SAT: Maximum score for subscale is 18, lower scores indicate decreased satisfaction with medicine

^i^CD-RISC10: mean score 31 with lower scores indicating less resilience

^j^SCS-R: Maximum score 120 with lower scores indicating less social connectedness

In addition, for each measure with a reported reference range (Emotional Exhaustion, Depersonalization, Stress, and Sleepiness), we determined the proportion of residents that exceeded the reference range [17–20,23].

## Results

**Figure 1.**
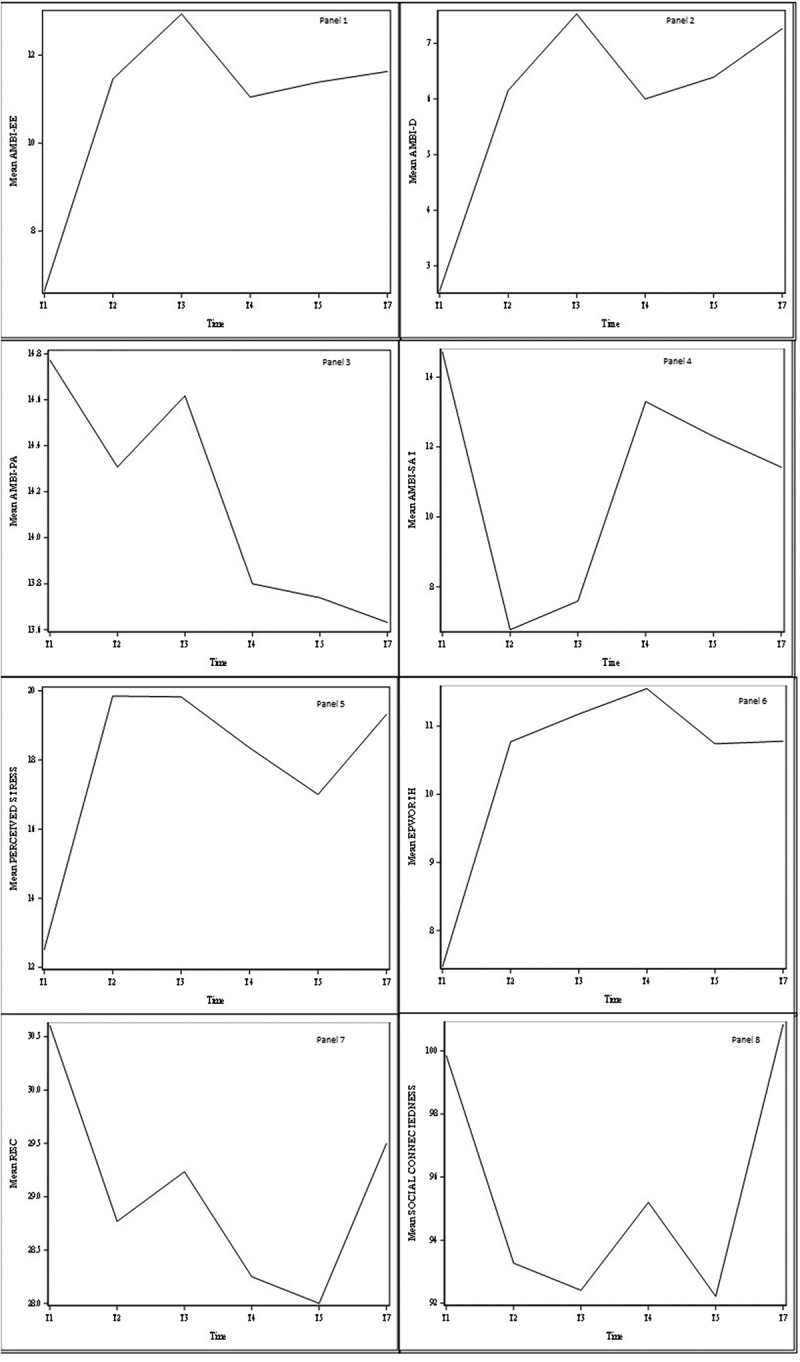
Trends in survey metrics over time.

The cohort baseline demographics, hospital characteristics, and residency training schedule are presented in Table 1. The surveys were provided to residents 6 times. At least 4 of the 6 survey distributions were completed by 23/33 trainees (69.7%) (Table 2). Three subjects completed surveys only at T1 and are not included in comparison statistical models. We defined each survey’s best score as the most desirable outcome and its worst score as its least desirable outcome. The data are summarized in Figure 1 and Tables 3–5.

**Table 4.** Differences between worst score and T7.

Survey^a,b^	Worst Score	Ending Timepoint	Least Squared Mean Difference between worst score and T7	Standard Error	Adjusted p-value^c^
AMBI-EE	T3	T7	−0.32	0.62	0.9952
AMBI-D	T3	T7	−0.14	0.64	0.9999
PSS	T2	T7	−0.03	1.79	1.0000
ESS	T4	T7	0.61	0.78	0.9675
AMBI-PA	T7	T7	NA	NA	NA
AMBI-SAT	T2	T7	−5.03	0.89	0.0000
CD-RISC10	T5	T7	−1.71	1.34	0.7947

^a^See text for abbreviations and descriptions; References [9–11,14–22]

^b^SCS-R not included because T7 mean score was higher than baseline.

^c^Using Tukey-Kramer method

**Table 5.** Prevalence of survey scores suggesting resident impairment.

**5a. All surveys (Prevalence of scores worse at follow-up than at baseline)^a^**					
**Survey^b^**	**TIMEPOINT**				
	**T2**	**T3**	**T4**	**T5**	**T7**
AMBI-EE	88.5%	94.1%	80.0%	69.6%	84.2%
AMBI-D	80.8%	94.1%	85.0%	87.0%	89.5%
PSS	88.5%	94.1%	90.0%	91.3%	89.5%
ESS	80.8%	88.2%	90.0%	78.3%	89.5%
AMBI-PA	46.2%	41.2%	65.0%	65.2%	52.6%
AMBI-SAT	96.2%	94.1%	65.0%	69.6%	73.7%
CD-RISC10	57.7%	70.6%	65.0%	69.6%	68.4%
SCSR	73.1%	76.5%	65.0%	78.3%	73.7%
**5b. Surveys with references ranges (Prevalence of scores beyond reference range)^c^**					
**Survey (Reported Reference Range)**	**TIMEPOINT**				
	**T2**	**T3**	**T4**	**T5**	**T7**
AMBI-EE (>9)	76.9%	82.4%	65.0%	69.6%	78.9%
AMBI-D (>6)	34.6%	58.8%	40.0%	39.1%	42.1%
PSS (>13)	84.6%	88.2%	75.0%	69.6%	68.4%
ESS (>10)	57.7%	47.1%	65.0%	52.2%	52.6%

^a^Proportion of competed surveys at each timepoint that were worse than at baseline (T1)

^b^See text for abbreviations and descriptions. References [9–11,14–22]

^c^See references [17–20,23] for reported reference ranges

See text for abbreviations and descriptions; References [9–11] and [14–23] for original survey details and reference ranges. Panel 1, AMBI-EE: maximum score 18; higher scores indicate feeling more emotional exhaustion.Panel 2, AMBI-D: maximum score 18; higher scores indicate feeling more depersonalization.Panel 3, AMBI-PA: maximum score 18; lower scores indicate feeling less personal accomplishment.Panel 4, AMBI-SAT: maximum score 18; lower scores indicate decreased satisfaction with medicine.Panel 5, Perceived Stress Scale: 0–13 low stress, 14–26 moderate stress, 27–40 high stress.Panel 6, Epworth Sleepiness Scale: 0–10 normal, 11–12 mild sleepiness, 13–15 moderate sleepiness, 16–24 severe sleepiness. Panel 7, CD-RISC10: mean score 31; lower scores indicate less resilience.Panel 8, SCS-R: maximum score 120; lower scores indicate less social connectedness.

### Emotional exhaustion (AMBI-EE)

The cohort baseline (T1) mean score was 6.64 (best). The worst score was at T3 (mean 12.94), improved slightly at T4, and declined again at T5 and T7 (Figure 1, Panel 1). The change from T1 to every timepoint was statistically significant (Table 3).

### Depersonalization (AMBI-D)

Mean AMBI-D scores tracked much like AMBI-EE (Figure 1, Panel 2). The cohort mean best score was 2.55 at T1 and the worst score was 7.56 at T3. Mean score improved slightly at T4 but worsened again at T5 and T7. The change from T1 to every timepoint was statistically significant (Table 3).

### Personal accomplishment (AMBI-PA)

The cohort mean best score was at T1 (14.77). As with other surveys, scores worsened as residency progressed (Figure 1, Panel 3). At T3, when AMBI-EE and AMBI-D were at their worst, AMBI-PA mean score was not statistically different from baseline (p = 0.4535) (Table 3). At all other times, AMBI-PA was significantly lower than baseline. The worst score was at T7, shortly before completion of residency training.

### Satisfaction with medicine (AMBI-SAT)

The cohort mean best score was 14.73 at T1. The mean score decreased to its nadir at T2 (6.77), slowly increased, but never returned to baseline (Figure 1, Panel 4). Change from T1 was statistically significant at each timepoint. The significant increase from T2 to T7 (p = 0.0000) suggests some recovery occurred (Table 4).

### Perceived stress (PSS)

The cohort mean best PSS score was 12.52 at T1. Changes in PSS were similar to AMBI-EE and AMBI-D. The 2 worst scores, 19.85 and 19.82 (moderate stress) were at T2 and T3, respectively. These values improved slightly at T4 and T5, and worsened again at T7 (Figure 1, Panel 5). The change from T1 to every timepoint was statistically significant (Table 3).

### Fatigue (ESS)

Mean cohort ESS at T1 was normal (7.47, best). It was >10 at all other timepoints, peaking at T4 (11.55) (Figure 1, Panel 6). Despite some improvement at T7, ESS remained worse than at T1, demonstrating persistent sleepiness, a proxy for fatigue.

### Resilience (CD-RISC10)

The cohort mean best score was at T1 (30.61). Resilience steadily decreased, reaching its worst score at T5 (28.0) (Figure 1, Panel 7). Small improvements at T3 and T7 were not statistically different from T5. Mean scores at T2 through T5 were significantly worse than T1; T1 and T7 were not significantly different (p = 0.3172) (Table 3).

### Social connectedness (SCS-R)

Trends in SCS-R showed a different pattern than other surveys. The mean at T1 was 99.85 and steadily declined to 92.22 at T5 (Figure 1, Panel 8). Each timepoint in comparison to T1 was statistically significant (Table 3). However, at T7, mean SCS-R was above the baseline mean, indicating full recovery.

There was little evidence of improvement from worst score to T7 for most measures. The worst AMBI-PA mean was at T7. Social connectedness was best at T7. Satisfaction with medicine improved significantly from T2 (worst) to T7 (Table 4).

Table 5 demonstrates aggregated individual survey responses. At least 65% of residents demonstrated worse scores than at baseline on 36/40 (90%) follow-up surveys (Table 5a). Table 5b shows the proportion of residents meeting criteria for the 4 measures with a reported reference range: emotional exhaustion; depersonalization; at least moderate stress; and at least mild excessive sleepiness [17–20,23]. Scores for emotional exhaustion and moderate stress exceeded the normal reference range at every follow-up timepoint for ≥65% of trainees.

## Discussion

This cohort of pediatric residents demonstrated burnout, stress, fatigue, became less socially connected, and became less resilient within 6 months of starting residency. Moreover, they rapidly developed a diminished sense of personal accomplishment and reduced satisfaction with medicine. These results indicate that shortly after entering residency, these successful, high-performing young physicians had experiences that were detrimental to their well-being.

Previous studies (not on pediatric residents) have also shown burnout early in training [24,25]. Specifically in our pediatric trainees, significant change from baseline existed in all surveys, at every study interval, except for CD-RISC (resilience) at T7 and AMBI-PA (personal achievement) at T3. With the exception of SCS-R (social connectedness), no other survey score returned to baseline (best) score. Using recently established cut-off values for burnout[23], the mean scores for this cohort met criteria for burnout on both emotional exhaustion (AMBI-EE) and depersonalization (AMBI-D) at every timepoint after PGY1 orientation. These worrisome mean scores were not merely skewed by a few outliers: ≥65% of residents had individual survey scores that were worse than baseline on 90% of follow-up surveys. Moreover, this cohort is not unique: 2 subsequent cohorts in our program have PGY1 AMBI-EE and AMBI-D scores that reflect what we describe here [26,27].

Most of the measures used followed similar patterns: worsening during residency with variable (but incomplete) improvement prior to graduation. Additionally, the most significant changes from baseline were generally at the T2-T4 surveys, corresponding to mid-PGY1 year through mid-PGY2 year (Table 2). Most pediatric residency programs similar in size to ours have similar schedules: significant inpatient time during PGY1, higher acuity/more critical care rotations during PGY2, and more elective time during PGY3. There might be a tendency to assume that it is the exposure to and experience with critically ill patients along with longer 24-hour shifts that generates burnout, however we identified evidence of burnout well before the PGY2 year. Though their schedule may be different than a pediatric trainee, this finding is consistent with previous studies on internal medicine residents that took place prior to work-hour changes that were mandated in 2011 [24,25,28]. The clinically challenging PGY2 year does not seem to be the cause of increasing levels or prevalence of burnout.

We expected more evidence of improvement in survey scores by the end of residency related to increased elective time and somewhat decreased inpatient loads for PGY3s, but we found improvement only in social connectedness and satisfaction with medicine. The factors associated with improvement or recovery in these realms remain to be identified. The strain and pressure of residency training might cause a cohort of residents to grow closer – few acquaintances outside of medicine are likely to understand what they are experiencing on a daily basis as their co-residents do[29]. In the months leading up to residency completion, PGY3 residents might also have more time to engage socially with each other, and they might try to solidify friendships prior to moving on to the next phase of their careers. Seeing light at the end of the training tunnel might also increase resiliency. On the other hand, there may be inherent stress in completion of residency, including starting a fellowship or a job or moving to a new city.

Clearly, the 2011 work hour restrictions [28] that governed the training of this cohort did not prevent the impairments we report. PGY1 schedules contained no 24-hour shifts and there were frequent periods of day/night shifts in place of some 24-hour calls during the PGY2 and PGY3 years. There is evidence that while sleep on night shifts is not significantly different from 24-hour calls, night shift schedules are associated with worse scores on measures of fatigue and burnout[29]. In addition, restricting work hours might diminish a resident’s sense of purpose and patient ‘ownership,’ and the perception of clinical responsibility might decrease when involvement in each patient’s care is time-limited by work rules.

### Potential limitations

This study took place at a single institution and we report a single cohort of pediatric residents. The study setting – a large, busy, high-pressure residency program – probably affected responses. Although the response rate was >50% at each timepoint and 69% of trainees completed surveys at least 4 times, there was not 100% participation after the initial distribution. There may be issues with the validity of surveys when they are used with groups not previously evaluated. The surveys we chose are commonly used to assess residents and/or medical students. The ESS measures sleepiness rather than fatigue but we believe those entities are closely linked in residents working long hours with hospitalized patients. The AMBI-SAT has not been validated as part of the MBI or AMBI; nevertheless, it has specific questions about career satisfaction that were relevant to this project and add to our understanding of the 3-year experience of this cohort.

Generalization of results to all pediatric residency programs should be made with caution, but recent data from a multi-center cross-sectional study of pediatric residents surveyed annually during the 3 years of our study showed that burnout was prevalent amongst pediatric trainees nationwide [30,31]. Our program participated in that study. The core results of our present longitudinal study are remarkably similar to that cross-sectional study despite differences in study design, definitions, and survey instruments: burnout was prevalent, persistent, and associated with stress and fatigue (or sleepiness). Unfortunately, data are not available to assess possible effects of geography, as well as differences in program size, rotation schedules, time on inpatient versus outpatient rotations, on-call schedules, patient volume, and program leadership. While there may be specific aspects of our program that affect resident well-being, what we describe in this longitudinal single-center study is not unique to our program.

## Conclusion

Burnout is a significant problem in pediatric residency but is only one factor among many that are evidence of impaired resident well-being. In the high-stakes, high pressure environment of pediatric residency, trainees are pushed cognitively, emotionally, and physically. They often suffer burnout. Fatigue, stress, resilience, and social connectedness also worsen during residency. This damage occurs early in training and is still present when training is completed. Many of the traits that are valued in pediatricians, including compassion and altruism, may be suppressed in physicians who are burned out; the American Academy of Pediatrics has recognized burnout as a serious issue affecting the pediatric workforce [32].

The results of this longitudinal study and the recently published national cross-sectional data reinforce each other. Effective interventions must be identified and should be introduced early in training. Despite strategies intended to reduce burnout in individuals willing to try them [33–37], as yet there are no interventions proven to reduce burnout or increase resilience in large groups of residents. The data indicate that burnout is too common among pediatric residents to be the result of personal attributes. While there may be individual factors that impair or protect well-being [31], it is evident that systemic problems in residency training must be identified and addressed.

Rather than offering interventions intended to assist or protect individuals who try them, the data support modification of how residents are trained. Much like injury prevention efforts, developing preventive measures that are ‘built in’ to the system will be more effective than requiring potential victims to protect themselves and more appropriate than trying to repair damage after it has occurred [38]. Carefully crafted, systematic interventions should be developed and tested in an organized, controlled manner across a multitude of training programs. The goal must be to develop approaches to residency training that prevent psychological injury to trainees and improve well-being while producing future generations of knowledgeable, caring physicians.

## Supplementary Material

Supplemental Material
