# Supplementary material for: Natural history of burnout, stress, and fatigue in a pediatric resident cohort over three years
Source: Med Educ Online. 2020 Sep 8;25(1):1815386. doi: 10.1080/10872981.2020.1815386 (PMC7655030; doi:10.1080/10872981.2020.1815386)
Supplement: Supplemental Material [file ZMEO_A_1815386_SM7735.docx]

**Resident Survey *Required**

1. Your study ID number: *

2. Today's date:

3. Your age today:

4. In the last six months have you had any significant life events? ⃝ Yes ⃝ No

5. If so, how many?

6. If yes, do you consider the events to be positive or negative? |
Number positive:
Number negative:

7. Tell us about your social situation today. Mark only one oval.
⃝ Living alone
⃝ Living with a roommate (not a spouse or partner)
⃝ Living with a spouse or partner

8. Has this changed in the past 6 months? Mark only one oval. ⃝ Yes ⃝ No

9. If yes, please describe:

10. Marital Status: Mark only one oval.
⃝ Single
⃝ Engaged to be married
⃝ Married
⃝ Separated from spouse
⃝ Divorced
⃝ Other:

11. Has this changed in the past 6 months? Mark only one oval. ⃝ Yes ⃝ No

12. If yes, please describe:

13. Do you have a child/children at home? Mark only one oval. ⃝ Yes ⃝ No

14. If yes, how many?

15. What are their ages?

**Directions**: Following are a number of statements that reflect various ways in which we view ourselves. Rate the degree to which you agree or disagree with each statement using the following scale (1 = Strongly Disagree and 6 = Strongly Agree). There is no right or wrong answer. Do not spend too much time with any one statement and do not leave any unanswered.

1 = Strongly Disagree 2 = Disagree 3 = Mildly Disagree 4 = Mildly Agree 5 = Agree 6 = Strongly Agree

16. I feel comfortable in the presence of strangers. Mark only one oval.
⃝ 1 ⃝ 2 ⃝ 3 ⃝ 4 ⃝ 5 ⃝ 6

17. I am in tune with the world. Mark only one oval.
⃝ 1 ⃝ 2 ⃝ 3 ⃝ 4 ⃝ 5 ⃝ 6

18. Even among my friends, there is no sense of brother/sisterhood. Mark only one oval.
⃝ 1 ⃝ 2 ⃝ 3 ⃝ 4 ⃝ 5 ⃝ 6

19. I fit in well in new situations. Mark only one oval.
⃝ 1 ⃝ 2 ⃝ 3 ⃝ 4 ⃝ 5 ⃝ 6

20. I feel close to people. Mark only one oval.
⃝ 1 ⃝ 2 ⃝ 3 ⃝ 4 ⃝ 5 ⃝ 6

21. I feel disconnected from the world around me. Mark only one oval.
⃝ 1 ⃝ 2 ⃝ 3 ⃝ 4 ⃝ 5 ⃝ 6

22. Even around people I know, I don’t feel that I really belong. Mark only one oval.
⃝ 1 ⃝ 2 ⃝ 3 ⃝ 4 ⃝ 5 ⃝ 6

23. I see people as friendly and approachable. Mark only one oval.
⃝ 1 ⃝ 2 ⃝ 3 ⃝ 4 ⃝ 5 ⃝ 6

24. I feel like an outsider. Mark only one oval.
⃝ 1 ⃝ 2 ⃝ 3 ⃝ 4 ⃝ 5 ⃝ 6

25. I feel understood by the people I know Mark only one oval.
⃝ 1 ⃝ 2 ⃝ 3 ⃝ 4 ⃝ 5 ⃝ 6

26. I feel distant from people Mark only one oval.
⃝ 1 ⃝ 2 ⃝ 3 ⃝ 4 ⃝ 5 ⃝ 6

27. I am able to relate to my peers Mark only one oval.
⃝ 1 ⃝ 2 ⃝ 3 ⃝ 4 ⃝ 5 ⃝ 6

28. I have little sense of togetherness with my peers Mark only one oval.
⃝ 1 ⃝ 2 ⃝ 3 ⃝ 4 ⃝ 5 ⃝ 6

29. I find myself actively involved in people’s lives Mark only one oval.
⃝ 1 ⃝ 2 ⃝ 3 ⃝ 4 ⃝ 5 ⃝ 6

30. I catch myself losing a sense of connectedness with society Mark only one oval.
⃝ 1 ⃝ 2 ⃝ 3 ⃝ 4 ⃝ 5 ⃝ 6

31. I am able to connect with other people Mark only one oval.
⃝ 1 ⃝ 2 ⃝ 3 ⃝ 4 ⃝ 5 ⃝ 6

32. I see myself as a loner Mark only one oval.
⃝ 1 ⃝ 2 ⃝ 3 ⃝ 4 ⃝ 5 ⃝ 6

33. I don’t feel related to most people Mark only one oval.
⃝ 1 ⃝ 2 ⃝ 3 ⃝ 4 ⃝ 5 ⃝ 6

34. My friends feel like family Mark only one oval.
⃝ 1 ⃝ 2 ⃝ 3 ⃝ 4 ⃝ 5 ⃝ 6

35. I don’t feel I participate with anyone or any group Mark only one oval.
⃝ 1 ⃝ 2 ⃝ 3 ⃝ 4 ⃝ 5 ⃝ 6

**Directions:** How likely are you to doze off or fall asleep in the following situations, in contrast to just feeling tired? This refers to your usual way of life recently. Even if you haven’t done some of these things recently, try to figure out how they would have affected you.

Use the following scale to choose the most appropriate number for each situation:

0=no chance of dozing 1=slight chance of dozing 2=moderate chance of dozing 3=high chance of dozing

It is important that you answer each item as best as you can.

36. Sitting and reading Mark only one oval.
⃝ 0 ⃝ 1 ⃝ 2 ⃝ 3

37. Watching TV Mark only one oval.
⃝ 0 ⃝ 1 ⃝ 2 ⃝ 3

38. Sitting inactive in a public place (e.g., a theater or a meeting) Mark only one oval.
⃝ 0 ⃝ 1 ⃝ 2 ⃝ 3

39. As a passenger in a car for an hour without a break Mark only one oval.
⃝ 0 ⃝ 1 ⃝ 2 ⃝ 3

40. Lying down to rest in the afternoon when circumstances permit Mark only one oval.
⃝ 0 ⃝ 1 ⃝ 2 ⃝ 3

41. Sitting and talking to someone Mark only one oval.
⃝ 0 ⃝ 1 ⃝ 2 ⃝ 3

42. Sitting quietly after a lunch without alcohol Mark only one oval.
⃝ 0 ⃝ 1 ⃝ 2 ⃝ 3

43. In a car, while stopped for a few minutes in traffic Mark only one oval.
⃝ 0 ⃝ 1 ⃝ 2 ⃝ 3

**Directions**: Please indicate how much you agree with the following statements as they apply to you over the last month. If a particular situation has not occurred recently, answer how you think you would have felt.

0 = Not at all true 1 = Rarely true 2 = Sometimes true 3 = Often true 4 = True nearly all the time

44. I am able to adapt when changes occur. Mark only one oval.
⃝ 0 ⃝ 1 ⃝ 2 ⃝ 3 ⃝ 4

45. I can deal with whatever comes my way. Mark only one oval.
⃝ 0 ⃝ 1 ⃝ 2 ⃝ 3 ⃝ 4

46. I try to see the humorous side of things when I am faced with problems. Mark only one oval.
⃝ 0 ⃝ 1 ⃝ 2 ⃝ 3 ⃝ 4

47. Having to cope with stress can make me stronger. Mark only one oval.
⃝ 0 ⃝ 1 ⃝ 2 ⃝ 3 ⃝ 4

48. I tend to bounce back after illness, injury, or other hardships Mark only one oval.
⃝ 0 ⃝ 1 ⃝ 2 ⃝ 3 ⃝ 4

49. I believe I can achieve my goals, even if there are obstacles. Mark only one oval.
⃝ 0 ⃝ 1 ⃝ 2 ⃝ 3 ⃝ 4

50. Under pressure, I stay focused and think clearly. Mark only one oval.
⃝ 0 ⃝ 1 ⃝ 2 ⃝ 3 ⃝ 4

51. I am not easily discouraged by failure. Mark only one oval.
⃝ 0 ⃝ 1 ⃝ 2 ⃝ 3 ⃝ 4

52. I think of myself as a strong person when dealing with life’s challenges and difficulties. Mark only one oval.
⃝ 0 ⃝ 1 ⃝ 2 ⃝ 3 ⃝ 4

53. I am able to handle unpleasant or painful feelings like sadness, fear, and anger. Mark only one oval.
⃝ 0 ⃝ 1 ⃝ 2 ⃝ 3 ⃝ 4

**Directions:** The questions in this scale ask you about your feelings and thoughts during the last month. In each case, you will be asked to indicate how often you felt or thought a certain way.

0 = Never 1 = Almost Never 2 = Sometimes 3 = Fairly Often 4 = Very Often

54. In the last month, how often have you been upset because of something that happened unexpectedly? Mark only one oval.
⃝ 0 ⃝ 1 ⃝ 2 ⃝ 3 ⃝ 4

55. In the last month, how often have you felt that you were unable to control the important things in your life? Mark only one oval.
⃝ 0 ⃝ 1 ⃝ 2 ⃝ 3 ⃝ 4

56. In the last month, how often have you felt nervous and “stressed”? Mark only one oval.
⃝ 0 ⃝ 1 ⃝ 2 ⃝ 3 ⃝ 4

57. In the last month, how often have you felt confident about your ability to handle your personal problems? Mark only one oval.
⃝ 0 ⃝ 1 ⃝ 2 ⃝ 3 ⃝ 4

58. In the last month, how often have you felt that things were going your way? Mark only one oval.
⃝ 0 ⃝ 1 ⃝ 2 ⃝ 3 ⃝ 4

59. In the last month, how often have you found that you could not cope with all the things that you had to do? Mark only one oval.
⃝ 0 ⃝ 1 ⃝ 2 ⃝ 3 ⃝ 4

60. In the last month, how often have you been able to control irritations in your life? Mark only one oval.
⃝ 0 ⃝ 1 ⃝ 2 ⃝ 3 ⃝ 4

61. In the last month, how often have you felt that you were on top of things? Mark only one oval.
⃝ 0 ⃝ 1 ⃝ 2 ⃝ 3 ⃝ 4

62. In the last month, how often have you been angered because of things that were outside of your control? Mark only one oval.
⃝ 0 ⃝ 1 ⃝ 2 ⃝ 3 ⃝ 4

63. In the last month, how often have you felt difficulties were piling up so high that you could not overcome them? Mark only one oval.
⃝ 0 ⃝ 1 ⃝ 2 ⃝ 3 ⃝ 4

**Directions**: Here are 12 statements of job-related feelings. Please read each statement carefully and decide if you ever feel this way about your job. If you have never had this feeling, choose the number “0” (zero) from the drop down menu. If you have had this feeling, indicate how often you feel it by choosing the number (from 1 to 6) that best describes how frequently you feel that way. An example is shown below.

How often: 0 = Never 1 = A few times a year or less 2 = Once a month or less 3 = A few times a month 4 = Once a week 5 = A few times a week 6 = Every day

Statement: I feel depressed at work. If you never feel depressed at work, you would choose the number “0” (zero) under the heading “How Often.” If you rarely feel depressed at work (a few times a year or less), you would choose the number “1.” If your feelings of depression are fairly frequent (a few times a week but not daily), you would choose the number “5.”

64. I deal very effectively with the problems of my patients. Mark only one oval.
⃝ 0 ⃝ 1 ⃝ 2 ⃝ 3 ⃝ 4 ⃝ 5 ⃝ 6

65. I feel I treat some patients as if they were impersonal objects. Mark only one oval.
⃝ 0 ⃝ 1 ⃝ 2 ⃝ 3 ⃝ 4 ⃝ 5 ⃝ 6

66. I feel emotionally drained from my work. Mark only one oval.
⃝ 0 ⃝ 1 ⃝ 2 ⃝ 3 ⃝ 4 ⃝ 5 ⃝ 6

67. I feel fatigued when I get up in the morning and have to face another day on the job. Mark only one oval.
⃝ 0 ⃝ 1 ⃝ 2 ⃝ 3 ⃝ 4 ⃝ 5 ⃝ 6

68. I’ve become more callous toward people since I took this job. Mark only one oval.
⃝ 0 ⃝ 1 ⃝ 2 ⃝ 3 ⃝ 4 ⃝ 5 ⃝ 6

69. I feel I’m positively influencing other people’s lives through my work. Mark only one oval.
⃝ 0 ⃝ 1 ⃝ 2 ⃝ 3 ⃝ 4 ⃝ 5 ⃝ 6

70. Working with people all day is really a strain for me. Mark only one oval.
⃝ 0 ⃝ 1 ⃝ 2 ⃝ 3 ⃝ 4 ⃝ 5 ⃝ 6

71. I don’t really care what happens to some patients. Mark only one oval.
⃝ 0 ⃝ 1 ⃝ 2 ⃝ 3 ⃝ 4 ⃝ 5 ⃝ 6

72. I feel exhilarated after working closely with my patients. Mark only one oval.
⃝ 0 ⃝ 1 ⃝ 2 ⃝ 3 ⃝ 4 ⃝ 5 ⃝ 6

73. I think of giving up medicine for another career. Mark only one oval.
⃝ 0 ⃝ 1 ⃝ 2 ⃝ 3 ⃝ 4 ⃝ 5 ⃝ 6

74. I reflect on the satisfaction I get from being a doctor. Mark only one oval.
⃝ 0 ⃝ 1 ⃝ 2 ⃝ 3 ⃝ 4 ⃝ 5 ⃝ 6

75. I regret my decision to have become a doctor. Mark only one oval.
⃝ 0 ⃝ 1 ⃝ 2 ⃝ 3 ⃝ 4 ⃝ 5 ⃝ 6

Thank you for completing this survey!
